# Regressive Autism Spectrum Disorder: High Levels of Total Secreted Amyloid Precursor Protein and Secreted Amyloid Precursor Protein-α in Plasma

**DOI:** 10.3389/fpsyt.2022.809543

**Published:** 2022-03-08

**Authors:** Xiaoli Li, Ping Zhou, Qiu Li, Bin Peng, Yupeng Cun, Ying Dai, Hua Wei, Xiao Liu, Yang Yu, Zhiyang Jiang, Qiongli Fan, Yuping Zhang, Ting Yang, Jie Chen, Qian Cheng, Tingyu Li, Li Chen

**Affiliations:** ^1^Growth, Development, and Mental Health of Children and Adolescence Center, Children's Hospital of Chongqing Medical University, Chongqing, China; ^2^Chongqing Key Laboratory of Child Health and Nutrition, Chongqing, China; ^3^Ministry of Education Key Laboratory of Child Development and Disorders, Chongqing, China; ^4^China International Science and Technology Cooperation Base of Child Development and Critical Disorders, Chongqing, China; ^5^National Clinical Research Center for Child Health and Disorders, Chongqing, China; ^6^School of Public Health and Management, Department of Health Statistics, Chongqing Medical University, Chongqing, China; ^7^Department of Pediatrics, Xinqiao Hospital, Army Medical University, Chongqing, China

**Keywords:** autism spectrum disorder, regression, amyloid precursor protein, sAPPα, biomarkers

## Abstract

Autism spectrum disorder (ASD) is a complex neurodevelopmental disorder characterized by social communication difficulties, repetitive behaviors, and parochial interests. Individuals with regressive ASD (RA), a unique subtype, have poor outcomes. Moreover, there are currently no validated blood-based biomarkers for ASD, hindering early diagnosis and treatment. This study was the first to examine plasma levels of total secreted amyloid precursor protein (sAPPtotal), secreted amyloid precursor protein-α (sAPPα), and secreted amyloid precursor protein-β (sAPPβ) in children diagnosed with RA (*n* = 23) and compare them with the levels in age-matched children with non-regressive ASD (NRA) (*n* = 23) and typically developing (TD) controls (*n* = 23). We found that sAPPtotal and sAPPα levels were significantly higher in children with RA than in children with NRA or in TD controls. In contrast, no difference was observed in sAPPβ levels. In conclusion, increased plasma levels of sAPPtotal and sAPPα may be valuable biomarkers for the early identification of ASD regression. Prospective studies will be conducted using a larger sample to further investigate these differences.

## Introduction

Autism spectrum disorder (ASD) is a neurodevelopmental disorder that emerges in early childhood and is characterized by social communication difficulties, repetitive behaviors, and parochial interests (1). Since the first description by psychiatrist Dr. Sukhareva, ASD has evolved from a rare to a widespread disease, with a prevalence of ~1.85%, and has become a major public health problem affecting social and economic development (2, 3). Overall, ASD has a serious impact on individuals, families, and societies.

The etiology and phenotypes of ASD are heterogeneous and determined by a complex combination of genetics and the environment (4, 5). At present, the diagnosis of ASD depends on behavioral descriptions and characteristic observations (6), with the average age at diagnosis being 5 years old (7). According to one prospective study, children with ASD exhibit social abnormalities and stereotyped behaviors at 6 months, but these subtle changes are usually ignored by parents (8). Early screening and diagnosis of ASD are very challenging, and the American Academy of Pediatrics recommends that children be screened early and continuously tested before the age of 2 years (9). Although behavioral interventions can improve outcomes, there are no drugs that completely alleviate the symptoms of ASD (10, 11). Importantly, earlier and more frequent behavioral interventions for autism lead to better outcomes (11). Notably, individuals with regressive ASD (RA), a complex subtype of the ASD phenotype, consistently have poor outcomes (12, 13), which may be related to the fact that individuals with RA show poorer language development, more severe autism, and lower intellectual function than those with non-regressive ASD (NRA) (14) as well as to the neurological and pathological bases of regression. At present, RA is a hot research topic.

Although the complex phenotypic causes and pathogenesis of RA have been explored for more than a century, no conclusions have yet been drawn (15). Tan et al. reported that the incidence of RA is up to 30% and that it generally occurs at the age of 19.8 months. Among individuals with RA, 20% exhibit language regression, 40% present language/social regression, 30% show mixed regression, and 27% exhibit unspecified regression (16). A recent prospective report pointed out that information reported by parents is only the tip of the iceberg and that the actual incidence of RA is as high as 80% (8). It has also been reported that immune disorders or neuroinflammation may be involved in the etiology of regression (17, 18). The only evidence involves data from metabonomics and immunology studies of older children (19, 20), and there are few biomarkers in early childhood in RA. Therefore, major goals are identifying early specific biomarkers and diagnostic tools for ASD and its subtypes before core symptoms and regression emerge.

Previous studies have identified 206 autism-susceptibility genes that converge on the amyloid precursor protein (APP) metabolic pathway (21). APP protein is a glycoprotein secreted by glial cells and neurons that promotes neuronal proliferation and migration, cell adhesion, and synapse formation (22, 23). In the non-amyloidogenic pathway, APP is cleaved by α- and γ-secretase liberates secreted APP-α (sAPPα) and p3 peptide (24). However, if APP is initiated by β- and γ-secretase, then secreted APP-β (sAPPβ) and neurotoxic Aβ peptides are generated, which are often involved in Alzheimer's disease (AD) and neurodegeneration (25–27). Notably, if APP is cleaved by γ-secretase at the C-terminus of the Aβ domain, secreted APP-γ (sAPPγ) may be released, and sAPPα, sAPPβ, and sAPPγ comprise total secreted APP (sAPPtotal) in human plasma (28). Plasma levels of sAPPtotal in individuals with severe and aggressive autism, a subtype of autism, are reportedly two or more times higher than those in children without autism (29). Moreover, sAPPα-overexpressing mice exhibit autism-like behavior and reduced social and exploratory behavior (30). Nevertheless, it remains unclear whether APP and its metabolites are involved in the pathophysiological pathways underlying other autism phenotypes.

RA is a form of neurodegeneration that emerges only in childhood (31), and clarifying the specific pathophysiological pathway associated with regression, which may help identify early biomarkers for autism and other neurodegenerative diseases (such as AD), would be a major advance in the field of neurodevelopment and neurodegeneration. Here, we investigated differences in the levels of sAPP isoforms in the plasma of children with and without RA. The identification of early specific biomarkers and associated pathophysiological pathways may guide the identification, diagnosis, and intervention before the emergence of the core symptoms and regression of ASD.

## Materials and Methods

### Ethics Statement

The study protocol was approved by the Ethics Committee of the Children's Hospital of Chongqing Medical University (2019; Institutional Review Board Study Approval No. 292) and registered in the Chinese Clinical Trial Registry (ChiCTR) (registration number ChiCTR2000031194). The parents of all subjects provided written informed consent and their agreement for participation in our study. This study conformed to the Declaration of Helsinki.

### Participants and Blood Collection

In total, 69 children aged 1.75–5.08 years who were treated at the Pediatrics Department of Children's Hospital of Chongqing Medical University were examined in this case-control study. Children with ASD (*n* = 46) had a confirmed diagnosis of autism according to the clinical criteria in the Diagnostic and Statistical Manual of Mental Disorders, 5th edition (DSM-5), as diagnosed by a proficient clinical psychologist, developmental pediatrician, or child psychiatrist. Using the Childhood Autism Rating Scale (CARS), we further determined symptoms of ASD, and those with CARS scores ≥30 were included in the autism group (32). The judgment of ASD's regressive behavior in this study refers to the definition of the same type of research articles (14, 15, 33, 34), and interviews with the parents or caregivers on the child's development process. Notably, some children diagnosed with ASD initially show a period of apparently typical development followed by a considerable loss of previously established skills, a phenomenon termed “regression”. Regression is defined as the loss of one or more developmental skills in the areas of personal-social abilities, gross motor performance, and/or fine motor performance after those skills have been acquired and maintained for 3 months. For example, parents of an 18-month-old boy might be asked if the child used his index finger to indicate his needs. If the parents said the child used to but had stopped doing so, then they were asked when the ability had appeared and whether it had lasted more than 3 months before disappearing. If the parents answered yes, the child would be identified as having undergone regression in personal-social skills. Another type of regression is language regression, defined as the loss of more than five spoken words used communicatively in children over 18 months of age.

Exclusion criteria included participants comorbid with other developmental disorders or psychiatric diseases (e.g., Rett syndrome, cerebral palsy, chronic seizures, and other congenital diseases). Children with autism who had experienced regression and lost acquired skills or knowledge for at least 3 months were included in the RA group (*n* = 23, 3.16 ± 0.77 years old); age- and sex-matched children who did not experience regression were included in the NRA group (*n* = 23, 3.15 ± 0.74 years old). Among those who experienced regression, 12 exhibited language regression, four social regression, four mixed regression, and three another type of regression. Age- and sex-matched typically developing (TD) volunteers composed the control group (*n* = 23, 3.16 ± 0.88 years old) (Table 1). Blood samples were collected, separated, and stored in strict accordance with the requirements of the experiment. The collection tubes containing EDTA and blood were centrifuged at 4°C at 1,000 × g for 10 min, after which the top layer of plasma was carefully removed and transferred to an enzyme-free 1.5 ml Eppendorf tube. The plasma was centrifuged at 4°C for 12 min and divided into equal portions to store at −80°C for further analysis. It should be noted that the plasma aliquots underwent no more than 1 freeze cycle.

**Table 1:** Subject demographics.

	**Autism spectrum disorder subtype**			**Typically** **developing** **(*n* = 23)**
	**Total** **(*n* = 46)**	**Regression** **(*n* = 23)**	**No regression** **(*n* = 23)**	
Age (y) (mean ± SD)	3.17 ± 0.75	3.16 ± 0.77	3.15 ± 0.74	3.16 ± 0.88
Sex, *n* (%)				
Male	38 (82.60)	19 (82.60)	19 (82.60)	19 (82.60)

### Follow-Up

After all the non-regressive children underwent protein measurements, their developmental progress was investigated by telephone follow-up every 3 months until they reached the age of 36 months. Children who underwent regression during follow-up were defined as having regressive ASD. The follow-up telephone calls asked the following questions:

(1) Did the child have gross motor, fine motor, or personal-social skills that suddenly disappeared or failed to progress after being mastered and maintained for more than 3 months, as opposed to being lost within a short period after the abilities appeared? Examples of personal-social skills were the ability to put on clothes and shoes with the help of their parents and button their clothes. Examples of gross motor skills were the ability to ride tricycles, jump on one foot, stand on one foot 2 out of 3 times for 5 s, etc. Examples of fine motor skills were the ability to build eight-story towers, correctly choose the longer of two line segments three out of three times, copy a drawing of a cross, etc.
(2) Did the child lose more than five spoken words that had once been used communicatively? Examples include the ability to say one's name, understand three to four prepositions, and speak a pair of antonyms.

On the other hand, it is generally believed that regression that occurs after 3 years of age should be defined as childhood disintegrative disorder (CDD) (35, 36). To reduce confounding factors, we generally do not include children with ASD who experience regression after age 3.

### Analysis of Plasma sAPPtotal, sAPPα, and sAPPβ Levels

Concentrations of sAPPtotal, sAPPα, and sAPPβ were detected by ELISA, as described by Erickson et al. (37–39). Plasma sAPPtotal concentrations were measured in duplicate using a commercial ELISA kit (IBL, Gunma, Japan). A 50 μl volume of plasma was diluted with 300 μl enzyme immunoassay buffer and mixed evenly. A 100 μl volume of the mixture was added to wells precoated with capture monoclonal anti-human APP (R12A1) and incubated overnight at 4°C after covering it with a plate lid. After washing several times, HRP-conjugated monoclonal anti-human APP (R101A4) was added to all wells and incubated the precoated plate for 30 min at 4°C after covering it with a plate lid. After several washes and the addition of the chromogen substrate, the colorimetric signal was detected at 450 nm using a microplate reader (Thermo). A standard curve was prepared by using known amounts of recombinant human APP protein, and the concentration for unknown samples was read from the standard curve. Levels of the other secreted APP isoforms were similarly measured using ELISA kits (IBL, Gunma, Japan).

### Statistical Analysis

All data were analyzed by SPSS 25 and R language statistical analysis software, and the Shapiro–Wilk test was used to test the normality of all data sets. A descriptive analysis is presented as the means (standard deviation) or medians (interquartile ranges). The Friedman test was used to compare levels between groups; the Bonferroni *post-hoc* test was employed for *post-hoc* analyses between groups. Adjusted *p*-values <0.05 were considered statistically significant. Receiver operating characteristic (ROC) curve analysis was performed to define the discriminatory value of the sAPPtotal and sAPPα proteins to separate RA from NRA and TD. To verify the independent samples, we present the data in boxplots.

## Results

**Figure 1:**
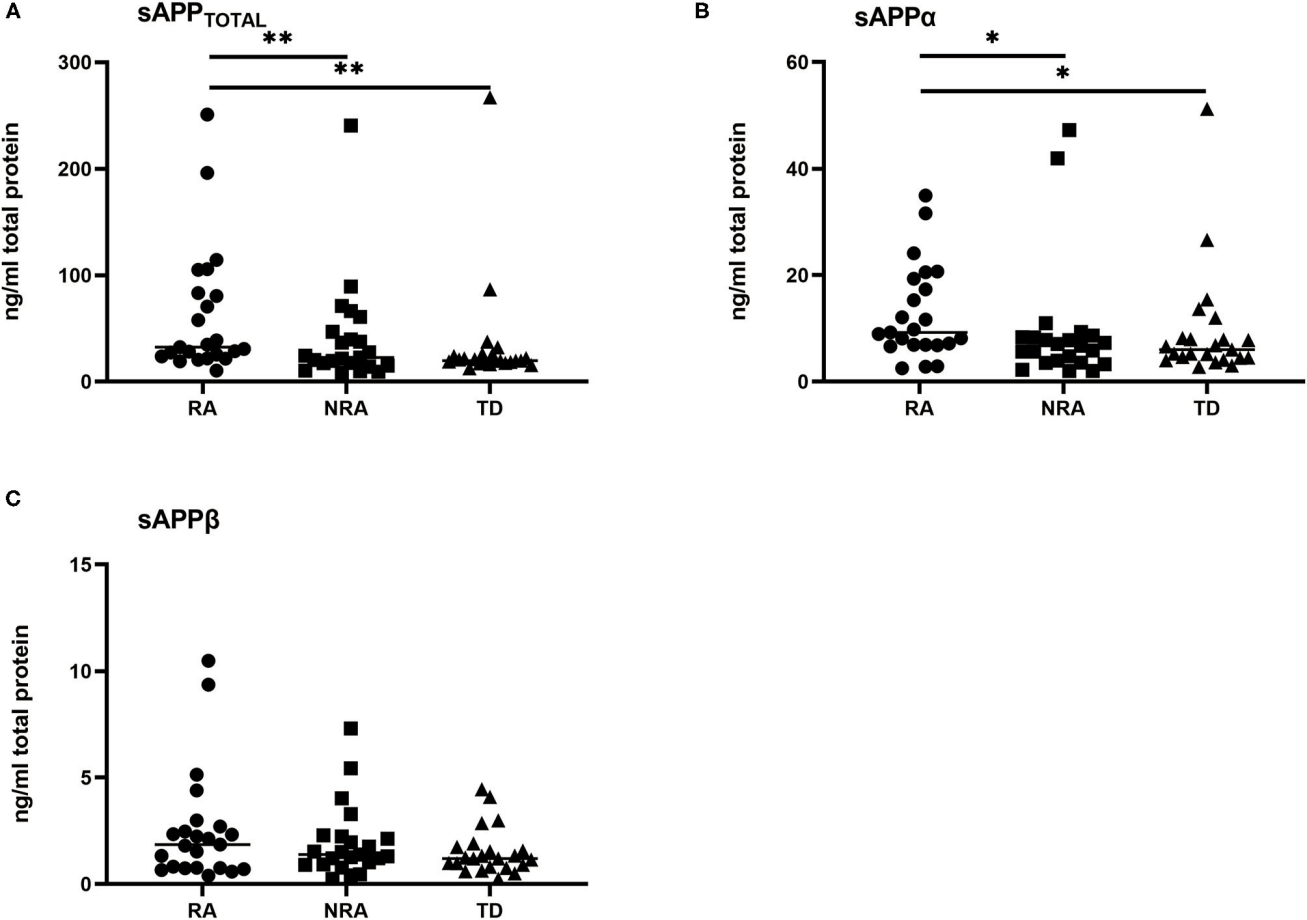
Levels of various secreted APP isoforms in different autism phenotypes and controls. **(A)** RA showed significantly elevated sAPPtotal levels compared with NRA (**p = 0.001) and TD (**p = 0.002). **(B)** RA had the highest sAPPα levels compared with NRA (*p = 0.024) and TD (*p = 0.024). **(C)** No significant difference was found in the sAPPβ levels of any two of the three groups. The Friedman test was used to calculate *p*-values, with Bonferroni *post-hoc* test correction.

In our study, the sAPPtotal and sAPPα levels were significantly higher in the RA group than in the NRA and TD groups. Levels of sAPPtotal (Figure 1A) and sAPPα (Figure 1B) were different among diagnoses: TD < NRA < RA. In contrast, no differences in sAPPβ levels were observed in any two of the three groups. Individual results are presented in Figure 1. sAPPtotal levels were significantly higher in the RA group than in the NRA group (*p* = 0.001) or the TD group (*p* = 0.002). Interestingly, no significant differences were observed between the NRA and TD groups. Furthermore, RA showed higher sAPPα levels than NRA (*p* = 0.024), with no significant differences between NRA and TD. More remarkably, there were no differences in the sAPPβ levels of any two of the three groups (*p* > 0.05).

**Figure 2:**
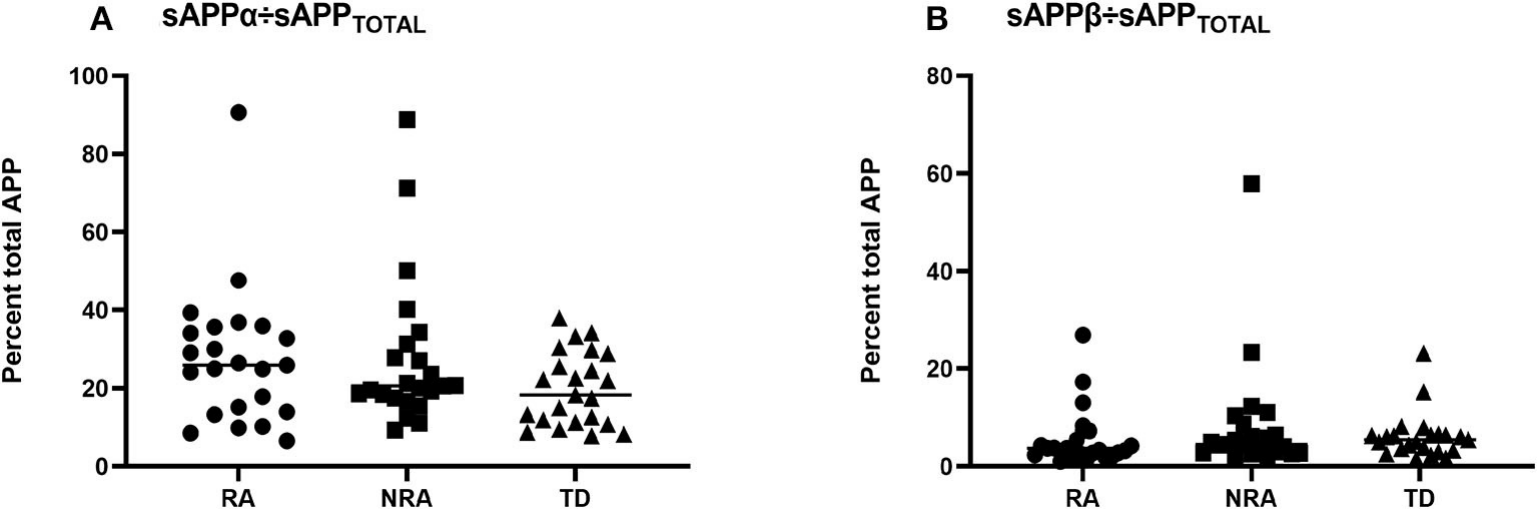
Ratios of the levels of different secreted APP isoforms in children with ASD and controls. To further explore differences in sAPPα, sAPPβ, and sAPPtotal levels, the **(A)** sAPPα/sAPPtotal ratio and **(B)** sAPPβ/sAPPtotal ratio were measured for each group, and there was no difference in any pairwise comparison of the three groups (*p* > 0.05). The Friedman test was used to calculate *p*-values, with Bonferroni *post-hoc* test correction.

To better characterize the balance between the amyloidogenic and non-amyloidogenic pathways in autism, sAPPα/sAPPtotal and sAPPβ/sAPPtotal ratios were measured for each group. However, no significant differences were observed between any two of the three groups (*p* > 0.05) (Figure 2).

**Figure 3:**
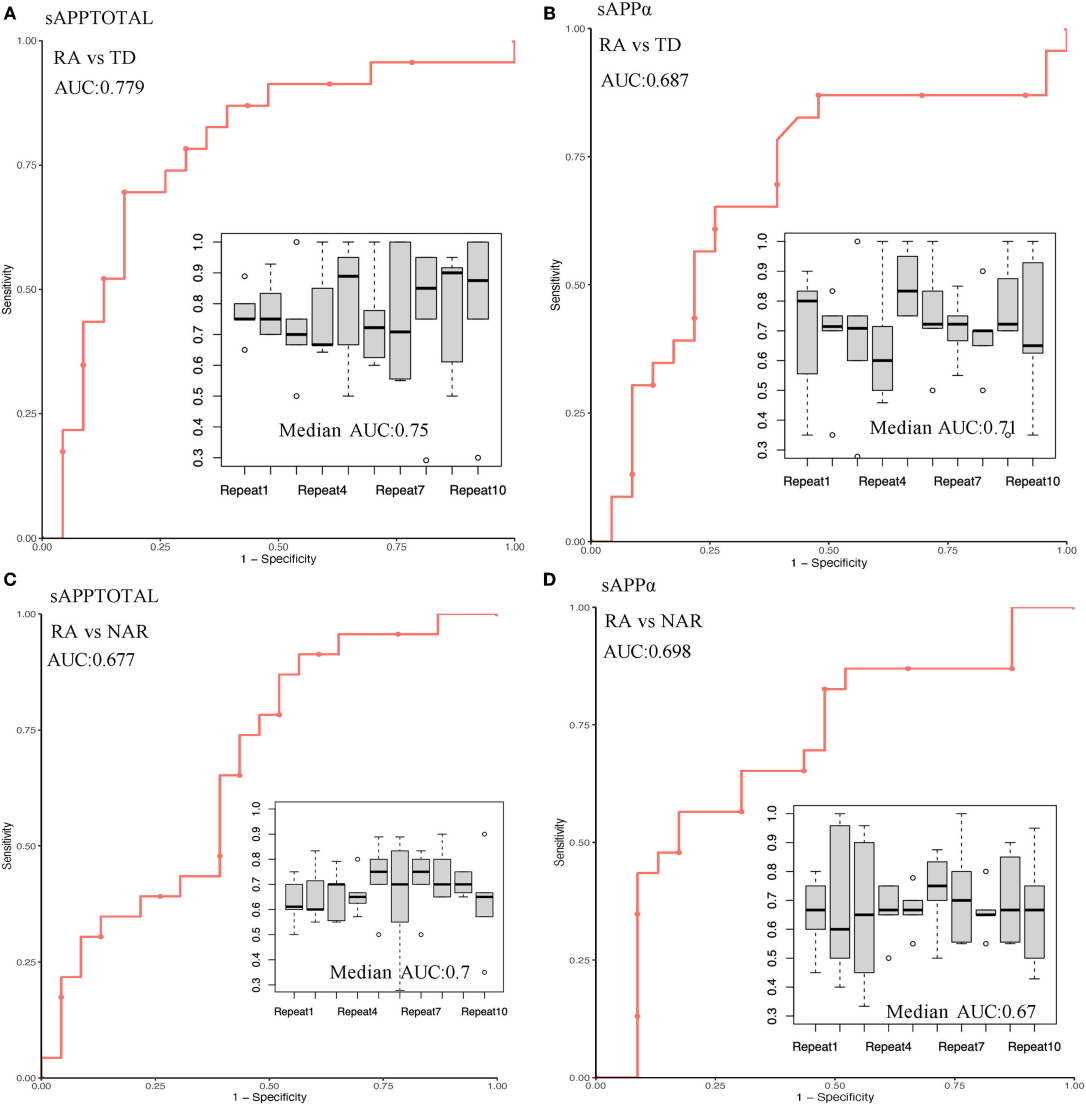
Prediction performance and independent validation of the sAPPtotal and sAPPα proteins in different groups. In the RA and TD groups, AUC measurements for the sAPPtotal and sAPPα protein to separate RA from TD were 0.779 **(A)** and 0.687 **(B)**, respectively. In the RA and NRA groups, AUC measurements for the sAPPtotal and sAPPα protein to separate RA from NRA were 0.677 **(C)** and 0.689 **(D)**, respectively. Repeated independent samples were used for ROC curve verification, and the data are represented by box graphs.

The use of sAPPtotal and sAPPα proteins as early specific biomarkers has predictive power to identify membership in the RA, NRA, and TD groups. Individual results are presented in Figure 3. The area under the ROC curve (AUC) of sAPPtotal for distinguishing RA from TD was 0.779, and the predictive AUC of independent sample validation was 0.75. ROC curve analysis revealed an AUC of 0.687 for the sAPPα protein to separate RA from TD, and the predictive AUC of independent sample validation was 0.71. In the RA and NRA groups, the AUC measurement for the sAPPtotal protein was 0.677 to separate RA from NRA, and the predictive AUC of independent sample validation was 0.7. ROC curve analysis revealed an AUC of 0.698 for the sAPPα protein to separate RA from NRA, and the predictive AUC of independent sample validation was 0.67.

## Discussion

Heller first defined regression as “dementia infantilis” in the early 20th century, and after more than a century, its mechanism is still unknown (15, 40). One epidemiological investigation found that the risk of AD in autistic patients is 5 times higher than that in individuals without developmental disabilities (41). Notably, the accumulation of Aβ in neurons is enhanced in the brains of idiopathic and dup15q11.2-q13 autism patients (42). APP metabolic pathways may be similar or different in individuals with RA and those with AD, but the relationship is not clear.

In contrast to AD, autism with regression of language, social or other abilities usually occurs in early childhood and gradually improves with age. Given the existence of neurodegenerative symptoms in early childhood in those with RA, we first examined differences between the levels of APP and its metabolites in children with and without RA. The results showed that plasma levels of sAPPtotal and sAPPα were higher in the RA group than in the NRA and TD groups, but there were no significant differences in sAPPβ levels between any two of the three groups.

High levels of sAPPα have been proven to be beneficial in the young adult brain, aged brain, and neurodegenerating brain (43). Indeed, studies on cultured neurons and animals have revealed that in the young brain, a high level of sAPPα aids in neurite outgrowth (44), increases synaptic density (45), stimulates neural stem cell proliferation (46) and differentiation (47), and enhances spatial memory and memory consolidation (48). Furthermore, during aging and neurodegeneration, a high level of sAPPα reduces oxidative stress and cell death (49), rescues synaptic plasticity and synapse number (50) and spatial memory (51), and reduces Aβ plaque deposition (52).

Nevertheless, excessive levels of sAPPα are harmful during the critical time window of brain development. During synaptic formation, excessive sAPPα reduces developmental spine pruning and impairs synaptic long-term depression (LTD), and changes in synaptic development and synaptic plasticity may result in ASD and memory impairment (53–55). In addition, excess sAPPα promotes the expression of inducible nitric oxide synthase (iNOS) in microglia, releasing glutamate and D-serine to cause neurotoxicity (56, 57). Moreover, an increased level of sAPPα during the critical window of development induces overdifferentiation of neural stem cells into astrocytes and enhances the brain immune response by disrupting the IL-6/gp130 pathway, leading to aberrant synaptic connections and brain damage (30).

However, it is worth noting where plasma sAPP, sAPPα, and sAPPβ come from. Based on the present study, we cannot determine whether they come from the brain or the gut. To date, no studies have confirmed whether the sAPPα protein originates from the gut or the brain. On the one hand, we consider that the sAPPα protein may come from the gut. In the last few years, the importance of gut microbiota impairment in the etiopathogenesis of pathologies such as autism and dementia has been raised (58). There has been an emerging interest in the possible role of the gut microbiota as a cofactor in the development of ASD; for example, serotonin is one of the possible links in the gut-brain–microbiome axis in ASD (59, 60). Some studies found that microbiome differences in ASD patients may be related to dietary preferences related to stereotyped behavior or narrow interest, thus weakening the association between ASD and intestinal microbiota (61). However, accumulating evidence has shown a link between alterations in the composition of the gut microbiota and both gastrointestinal and neurobehavioral symptoms in children with ASD (62). Furthermore, studies have shown that gut microbial dysbiosis may lead to the secretion of amyloid, which disturbs gastrointestinal permeability and the blood-brain barrier (BBB). In this way, it may induce neuronal injury and ultimately lead to neuronal death (63–65). However, there is not enough evidence to prove whether the sAPPα protein, as an amyloid precursor protein cleavage product, can cross the BBB and have harmful effects during critical periods of brain development, which will be a very interesting and valuable research direction. On the other hand, we consider that sAPPα proteins may leak from the BBB. Studies have shown that excess sAPPα can activate microglia and astrocytes (30, 57), which contribute to BBB dysfunction (66), but it is unclear whether the sAPPα protein will leak from the damaged BBB, which needs to be further verified in animal experiments and will be a very valuable direction for our subsequent research. In conclusion, with the limitations of the current sample, we were able to obtain only peripheral blood in the hope of finding early predictive markers; in our next step, we will verify these markers in animal experiments to explore the source of all sAPP proteins. Significantly, in our study, compared with the RA and TD groups, children with regressive autism had higher sAPPtotal and sAPPα levels. It may be valuable for the early identification of ASD regression.

RA, a distinct subtype of autism in which the non-amyloidogenic pathway may be preferentially active, may be the result of associated pathophysiological changes. In conclusion, we believe that an increase in sAPPα levels may reflect an early increase in the body's responsiveness but that an excessive increase in childhood may lead to neurotoxicity, aberrant synaptic connections, and brain damage. Therefore, inhibiting an abnormal increase in sAPPα may help prevent the regression process. Overall, sAPPtotal and sAPPα levels may serve as biomarkers for the early diagnosis and prognosis of RA.

Ray found that compared to other individuals (67), sAPPβ levels are decreased in individuals with severe autism; however, our study did not detect decreased levels of sAPPβ in children with RA. Notably, the average age of individuals with severe autism in Ray's study was 8.17 years, whereas the average age of children with RA in our study was 3.13 years. A potential explanation for the discrepancy is that the expression of APP and its metabolites differs during the various stages of childhood. It is not clear whether the activity of the toxic metabolic pathway that produces sAPPβ gradually declines with age, the level of sAPPα increases with age, and its physiological effect is beneficial. This hypothesis needs to be verified by prospective cohort studies with longer-term observations in the future.

## Limitations

There are limitations to our study. For example, children were included based on retrospective reports of parents, which may have affected our results. Therefore, a prospective study of children with RA might more accurately reveal the dynamic changes in the levels of APP and metabolites in these individuals.

## Conclusions

Clarifying the common neurobiological mechanisms of neurodevelopmental disorders and neurodegenerative disease may be useful in identifying early disease biomarkers. Importantly, we observed that plasma levels of sAPPα and sAPPtotal were elevated in early childhood in individuals with RA. Increased plasma levels of sAPPtotal and sAPPα may be secondary changes in response to another pathological change that might be the causal pathology. Our study may be valuable biomarkers for the early identification of ASD regression.

Prospective studies will be conducted using a larger sample to further investigate these differences.

## Data Availability Statement

The raw data supporting the conclusions of this article will be made available by the authors, without undue reservation.

## Ethics Statement

The studies involving human participants were reviewed and approved by the Ethics Committee of the Children's Hospital of Chongqing Medical University (approval number (2019) IRB (Study) No. 292). Written informed consent to participate in this study was provided by the participants' legal guardian/next of kin.

## Author Contributions

TL and LC conceived and designed the experiments. XLi, PZ, and QL performed the experiments. BP, YC, YD, HW, XLiu, YY, QF, YZ, ZJ, TY, JC, and QC contributed reagents, materials, analysis tools. XLi and LC wrote the paper. All authors contributed to the article and approved the submitted version.

## Funding

This study was supported by the Youth Innovation Team of Future Medical Support Plan of Chongqing Medical University (W0037), the Guangzhou Key Project Early diagnosis and treatment of autism spectrum disorders (202007030002), and the Guangdong Key Project Development of new tools for diagnosis and treatment of autism (2018B030335001).

## Conflict of Interest

The authors declare that the research was conducted in the absence of any commercial or financial relationships that could be construed as a potential conflict of interest.

## Publisher's Note

All claims expressed in this article are solely those of the authors and do not necessarily represent those of their affiliated organizations, or those of the publisher, the editors and the reviewers. Any product that may be evaluated in this article, or claim that may be made by its manufacturer, is not guaranteed or endorsed by the publisher.

## Abbreviations

ASD: autism spectrum disorder
RA: regressive autism
NRA: nonregressive autism
APP: amyloid precursor protein
sAPPα: secreted APP-α
sAPPβ: secreted APP-β
sAPPγ: secreted APP-γ
sAPPtotal: total secreted APP
Aβ: amyloid-β
AD: Alzheimer's disease
ChiCTR: Chinese Clinical Trial Registry
DSM-5: Diagnostic and Statistical Manual of Mental Disorders 5th edition
CARS: Childhood Autism Rating Scale
CDD: childhood disintegrative disorder
TD: typically developing
LTD: long-term depression
iNOS: inducible nitric oxide synthase
BBB: blood-brain barrier.
